# Longitudinal DNA methylation differences precede type 1 diabetes

**DOI:** 10.1038/s41598-020-60758-0

**Published:** 2020-02-28

**Authors:** Randi K. Johnson, Lauren A. Vanderlinden, Fran Dong, Patrick M. Carry, Jennifer Seifert, Kathleen Waugh, Hanan Shorrosh, Tasha Fingerlin, Brigitte I. Frohnert, Ivana V. Yang, Katerina Kechris, Marian Rewers, Jill M. Norris

**Affiliations:** 1 University of Colorado Anschutz Medical Campus, Division of Biomedical Informatics and Personalized Medicine, Aurora, CO USA; 2 Colorado School of Public Health, Department of Biostatistics and Informatics, Aurora, CO USA; 3 Barbara Davis Center for Diabetes, University of Colorado Anschutz Medical Campus, Aurora, CO USA; 4 Colorado School of Public Health, Department of Epidemiology, Aurora, CO USA; 5 National Jewish Health, Denver, CO USA

**Keywords:** DNA methylation, Type 1 diabetes, Epidemiology, Paediatric research

## Abstract

DNA methylation may be involved in development of type 1 diabetes (T1D), but previous epigenome-wide association studies were conducted among cases with clinically diagnosed diabetes. Using multiple pre-disease peripheral blood samples on the Illumina 450 K and EPIC platforms, we investigated longitudinal methylation differences between 87 T1D cases and 87 controls from the prospective Diabetes Autoimmunity Study in the Young (DAISY) cohort. Change in methylation with age differed between cases and controls in 10 regions. Average longitudinal methylation differed between cases and controls at two genomic positions and 28 regions. Some methylation differences were detectable and consistent as early as birth, including before and after the onset of preclinical islet autoimmunity. Results map to transcription factors, other protein coding genes, and non-coding regions of the genome with regulatory potential. The identification of methylation differences that predate islet autoimmunity and clinical diagnosis may suggest a role for epigenetics in T1D pathogenesis; however, functional validation is warranted.

## Introduction

Type 1 diabetes (T1D) is a chronic, autoimmune disease affecting approximately 40 million people worldwide^1^. Genetic susceptibility plays a major role in development of islet autoimmunity (IA), which is characterized by destruction of insulin-producing beta cells in the pancreas. Non-genetic factors also contribute to etiology of T1D since genetics cannot account for the rapidly increasing incidence, the high early-life disease discordance in genetically identical twins, nor the seasonal diagnosis patterns^2–4^. Inconsistent findings of commonly studied factors such as viral infections, infant diet, and gut microbial composition suggest that the triggering environmental risk factors vary by age, autoimmune phasing, or genotype^5–10^. Though no particular environmental exposure has been firmly established in pathogenesis of T1D, epigenetics has been proposed as a link between genetic susceptibility and environmental exposures that may account for some of the disease heterogeneity^11,12^.

Epigenetics generally refers to modifications to DNA that alter gene expression but not the DNA sequence, and includes DNA methylation and chromatin remodeling^13,14^. The most commonly studied epigenetic mechanism is DNA methylation, which involves the addition of a methyl group to a CpG site that can affect or reflect transcriptional activity^15^. For example, DNA methylation at specific genomic loci may be altered in response to environmental stimuli, maintained in daughter cells, and directly influence disease development by altering gene expression^16^. Alternately, DNA methylation may be altered as a consequence of disease progression, and therefore serve as a biomarker of pathogenesis.

Differences in DNA methylation levels and variability have been associated with T1D in previous case-control studies^17–21^. Given these epigenome-wide association studies were performed on individuals already diagnosed with diabetes, it has yet to be established whether methylation plays a role in the development of disease or whether it is merely a marker of the metabolic derangements associated with symptomatic (prevalent) diabetes.

**Figure 1:**
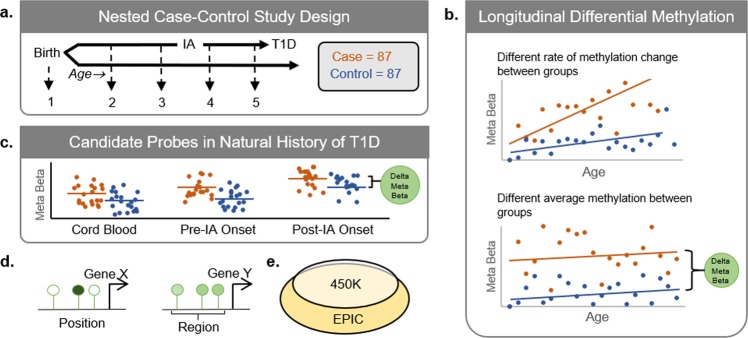
Study design and analytical approach to identify longitudinal differential methylation preceding type 1 diabetes. (**a**) We measured the proportion of DNA methylation (Beta, ranging 0 to 1) on one to five pre-disease samples for 87 cases (orange) and 87 frequency-matched controls (blue) from DAISY. Selected samples included cord blood (birth, 1), infancy between 9 and 16 months (2), the last disease-free sample prior to IA onset (3), the first sample after IA onset, when IA was first detected (4), and the last sample prior to T1D diagnosis (5). (**b**) We examined longitudinal differences in the rate of methylation change between cases and controls and the average methylation difference between cases and controls. Linear mixed models estimated the longitudinal difference in changing methylation, and the average longitudinal difference in methylation (change in the meta-analysis beta—“delta meta beta”) between T1D case and control groups. Results from probes measured on both 450 K and EPIC platforms were combined using a meta-analysis. (**c**) To examine the impact of preclinical autoimmunity at candidate probes, we examined cross-sectional differences in methylation at three important periods in the natural history of T1D: cord blood (1), pre-IA onset (2 or 3), and post-IA onset (4 or 5). Cross-sectional lookup analyses included 37 cases and 35 controls at cord blood, 43 cases and 44 controls at pre-IA onset, and 85 cases and 85 controls at post-IA onset. (**d**) Analyses were performed on both the position and region level. (**e**) The majority of probes on the 450 K array were also present on the EPIC array.

We measured DNA methylation in peripheral whole blood collected prior to onset of clinical T1D from individuals enrolled in the prospective Diabetes Autoimmunity Study in the Young (DAISY) cohort, which follows high-risk children for the development of IA and T1D. Measurements from up to five time points prior to diagnosis of disease for 87 cases and 87 frequency-matched controls allowed us to determine for the first time that pre-disease methylation differences are associated with later development of T1D (Fig. 1). Subjects were split into two sets for methylation quantification using the Infinium HumanMethylation450K Beadchip (“450 K”) and the Infinium MethylationEPIC Beadchip (“EPIC”). Using a meta-analysis of the two platforms, we identified longitudinal differential methylation that changed with age (differentially changing methylation positions and regions, DCMPs and DCMRs respectively) and longitudinal differential methylation that did not change with age (differentially methylated positions and regions, DMPs and DMRs respectively). Candidate probes representing DMPs and DMRs were consistent throughout the natural history of preclinical T1D, including before and after the onset of IA, though some were different at birth.

## Results

### DNA methylation in study population

For investigating pre-disease methylation in DAISY, we frequency-matched 87 T1D cases to autoantibody-negative controls based on age at seroconversion to IA, race-ethnicity and sample availability (Fig. 1). IA was defined by detection of at least one confirmed, persistent autoantibody to insulin, GAD65, IA-2, or ZnT8. Subjects were randomly split into two sets, keeping serial samples for each case and their frequency matched controls in the same set. We excluded cord blood samples from longitudinal statistical analyses since, by design, only children recruited into DAISY via newborn screening (~50%) were likely to have cord blood samples collected (see Methods).

For longitudinal analyses of genome-wide DNA methylation, set one included 184 samples from 84 subjects profiled on the 450 K platform (mean = 2.2 samples per subject). Set two included 211 samples from 90 subjects profiled on the EPIC platform (mean = 2.3 samples per subject). In both the 450 K and EPIC, subjects tended to be non-Hispanic white (Table 1). High-risk HLA genotype (HLA-DR3/4, DQB1*0302) was related to T1D in both sets. Cases developed T1D around age 10 years in both the 450 K and EPIC. Subjects and samples from the study population covered ages from infancy to adolescence, when the highest rates of new T1D diagnoses occur^22^. Data pre-processing and quality control were conducted in parallel on both platforms (Supplementary Fig. S1), including filtering to exclude probes on sex chromosomes, with SNPs in the CpG, or with low methylation range, which have poor reproducibility in epidemiological studies^23^ (see Methods). We retained good coverage of the genome despite the heavy filtering—the 199,243 probes that passed filtering criteria covered 68.6% of 450 K autosomal genes pre-filtering—due to high redundancy of multiple probes per gene on the Illumina platforms^24^. Normalized M-values were used for all subsequent statistical analyses, while Beta-values (scale of 0 to 1, proportion methylated) were used for biological interpretation in tables and figures^25^. For interpretation, we used functional annotation from Ensembl to identify the nearest gene to each probe.

**Table 1:** Characteristics of T1D cases and frequency-matched controls in the longitudinal DAISY nested case-control study.

	450 K		EPIC	
	Case	Control	Case	Control
	N = 42	N = 42	N = 45	N = 45
	n (%)			
Non-Hispanic White	40 (95.2)	39 (92.9)	37 (82.2)	37 (82.2)
Hispanic	2 (4.8)	3 (7.1)	7 (15.6)	7 (15.6)
Black	—	—	1 (2.2)	1 (2.2)
Male	23 (54.8)	28 (66.7)	20 (44.4)	24 (53.3)
High-Risk HLA (DR3/4, DQB1*0302)	20 (47.6)	5 (11.9)*	22 (48.9)	12 (26.7)*
	Mean (SD)			
Age at T1D diagnosis (years)	10.1 (4.8)	—	9.3 (4.4)	—

*Indicates significant difference between T1D cases and controls (p < 0.05).

### Longitudinal differentially changing methylation positions (DCMPs) and regions (DCMRs)

We used linear mixed models adjusted for sex and age to test differences in the rate of methylation change over time between T1D cases and controls by including an interaction term with age (see Methods). None of the 199,244 probes tested were DCMPs; the rate of methylation change (slope) did not significantly differ for the tested probes between T1D case and control groups at false discovery rate adjusted meta p-value < 0.1. Given that methylation may affect adjacent positions together, we also investigated differentially changing methylation regions (DCMRs). All 199,244 probes tested in DCMP analyses were combined into regions using the “comb-p” tool^26^. Regions that contained more than one probe and had a combined regional adjusted p-value < 0.1 were considered DCMRs. We identified 10 DCMRs (Supplementary Table S1), the majority of which were located near protein-coding genes.

**Figure 2:**
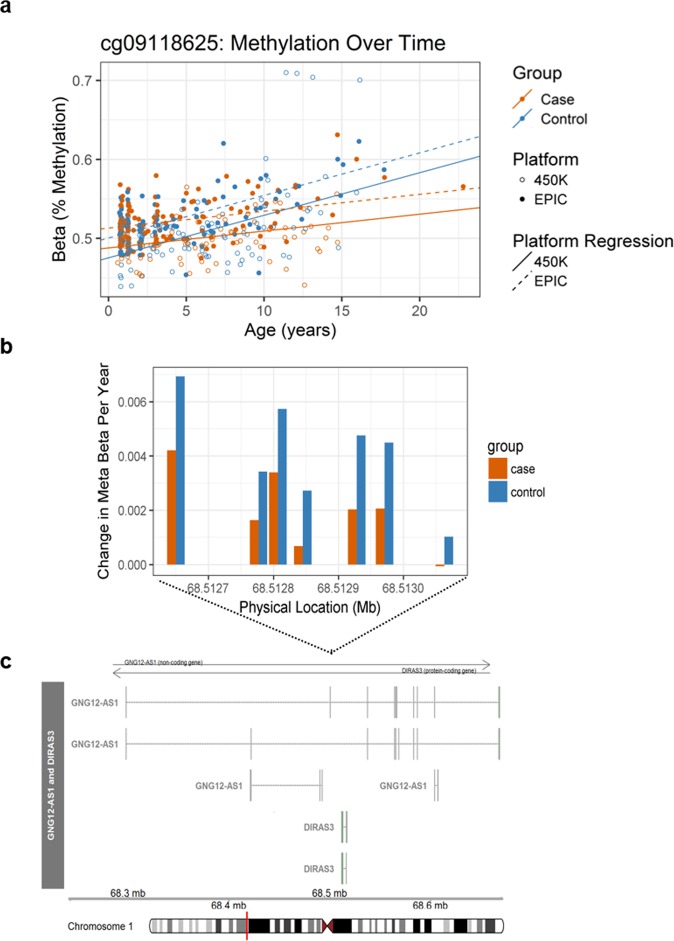
The most statistically significant of 10 differentially changing methylation regions (DCMRs) associated with development of T1D. (**a**) Methylation (%, Beta) in longitudinal samples from T1D cases (orange) and controls (blue) from the 450 K (open circle) and EPIC (solid circle) for the most statistically significant DCMP involved in the DCMR (cg09118625). The regression line shows average longitudinal methylation over time from the 450 K (solid) and EPIC (dotted) for each group. The meta estimated slope for cases = 1.2% and controls = 2.6%. (**b**) The change in meta beta per year is shown for T1D cases (orange) and controls (blue) across all seven probes in the DCMR on chromosome 1, located near both GNG12-AS1 and DIRAS3. (**c**) Structure of GNG12-AS1 and DIRAS3 isoforms from Ensembl are displayed, including: exons (boxes) and introns (lines). The arrow indicates the direction of transcription. The black box above the transcript structures represents the zoomed in region where the probes involved in the DCMR are located. The red line on the ideogram at the bottom shows on a chromosomal level where the DCMR is located.

For the most statistically significant DCMR (combined regional adjusted meta p-value = 6.72 × 10^−6^), methylation increased faster over time for controls than for T1D cases (0.4% average increase per year compared to 0.2% average increase per year, respectively). Figure 2a shows the change in methylation over time for the most individually statistically significant of seven probes in the region (cg09118625, case slope = 1.2%, control slope = 2.6% meta p-value = 9.72 × 10^−4^). The other six probes had similarly higher rate of methylation increase in controls compared to cases (Fig. 2b). This DCMR spanned the 5′UTR, introns, and coding sequence of the *GNG12-AS1* and *DIRAS3* genes on chromosome 1. The differential rate of methylation change in cases and controls for other DCMRs are provided in Supplementary Table S1.

### Longitudinal differentially methylated positions (DMPs)

**Figure 3:**
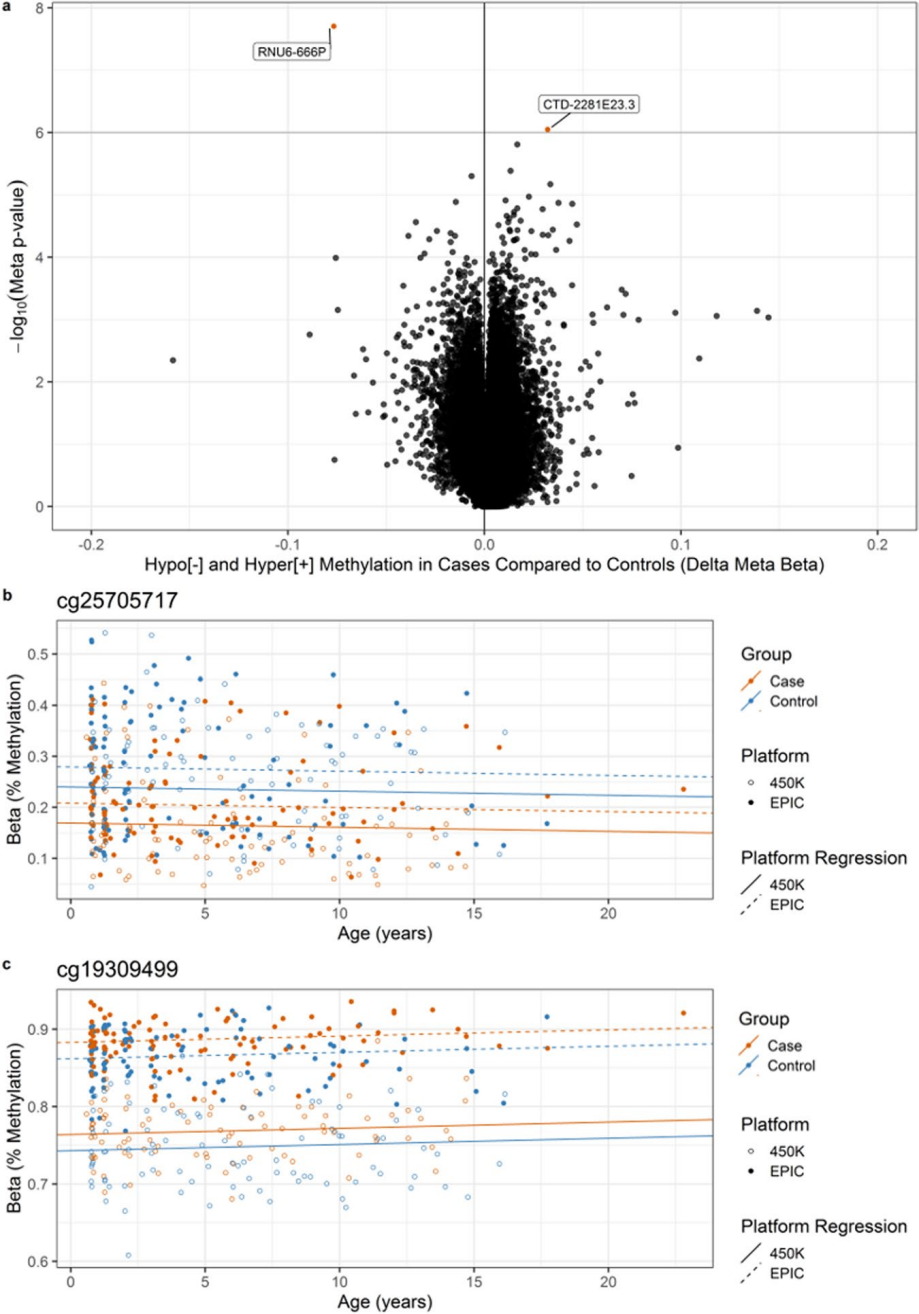
Differentially methylated positions (DMPs) longitudinally associated with development of T1D. (**a**) Volcano plot of meta-analysis results from 199,243 probes tested in the 450 K and EPIC using linear mixed models adjusted for sex and age. Candidate probes with adjusted meta p-value < 0.1 (orange) were considered DMPs, and annotated to the gene nearest the probe using Ensembl. (**b**,**c**) Methylation (%, Beta) in longitudinal samples from T1D cases (orange) and controls (blue) from the 450 K (open circle) and EPIC (solid circle) for the two DMPs. The regression line shows average longitudinal methylation over time from the 450 K (solid) and EPIC (dotted) for each group.

We next tested whether the average longitudinal methylation differed between T1D cases and controls using linear mixed models adjusted for sex and age. From 199,243 positions tested, we detected 2 DMPs where the average longitudinal methylation differed between case and control groups (false discovery rate adjusted meta p-value < 0.1, Fig. 3a). Our analytic pipeline minimized genomic inflation in both platforms, as determined by genomic inflation factors close to one (Supplementary Fig. S2).

The most statistically significant DMP (cg25705717, meta p-value = 1.97 × 10^−8^) also had the largest effect size, with an average −8.08% hypomethylation in T1D cases compared to controls (Fig. 3b). Cg25705717 was located on chromosome 10, approximately 4.6 kilobases from the nearest gene, which encodes a small nuclear RNA (*RNU6-666P*). The other DMP (cg19309499, meta p-value = 8.92 × 10^−7^) was characterized by 2.49% hypermethylation in T1D cases compared to controls (Fig. 3c). Cg19309499 was located on chromosome 8 near the long intergenic non-coding RNA *CTD-2281E23.3* and the gene *ERICH1-AS1*.

To summarize identified DNA methylation differences that preceded T1D, we tested for enrichment of genes from DMP analyses using missMethyl^27^. No GO terms (Supplementary Table S2) nor KEGG pathways (Supplementary Table S3) were significantly enriched by candidate genes, using positions with an unadjusted meta p-value < 0.001 (n = 299).

### DMP analysis robust to cell type composition adjustment

The proportion of different cell types (granulocytes, monocytes, CD4+ T cells, CD8+ T cells, CD19+ B cells) in un-sorted blood may reflect changing pathology due to the autoimmune process, rather than a confounding effect for which many DNA methylation studies account using statistical adjustment^28^. Like other studies of DNA methylation^29,30^, our primary methylation analyses did not include cell type adjustment due to this hypothesis. However, we did examine the relationship between methylation and T1D while adjusting for age, sex and cell type proportions using a linear mixed model. The resulting effect sizes were compared to those from the original analysis without adjusting for cell type proportions to determine its potential to attenuate results in DMP analyses.

Average longitudinal differences in methylation between T1D cases and controls identified in DMP analyses were robust to additional adjustment for cell type proportions (Supplementary Fig. S3). Cell type proportions estimated using the Houseman method^31^ were not statistically different between T1D cases and controls, using longitudinal mixed models adjusted for sex and age. Meta beta values for the T1D cases and controls were estimated using the same longitudinal model previously described plus adding the cell type proportions as covariates. The methylation difference between cases and controls (“delta meta beta”) changed minimally after cell type adjustment (median absolute difference = 5.4 × 10^−4^, IQR: 2.4 × 10^−4^ to 1.1 × 10^−3^).

### Longitudinal differentially methylated regions (DMRs)

The 199,243 probes tested in average longitudinal DMP analyses were combined into regions using comb-p; regions that contained more than one probe and had a combined regional FDR adjusted p-value < 0.1 were considered DMRs. We identified 28 DMRs from 63 regions (Supplementary Table S4). Each DMR contained between 2 and 7 probes and spanned 38 to 1216 base pairs on the genome. Fourteen DMRs (50%) had average hypermethylation in T1D cases compared to controls across all probes in the region, with effects ranging from 1.2% to 7.1%. The remaining 14 DMRs had average hypomethylation in T1D cases compared to controls across all probes in the region, with effects ranging from −0.4% to −4.2%. All probes within each DMR had consistent direction of effect—e.g.: all probes in a DMR had either hypermethylation or hypomethylation in cases compared to controls.

**Figure 4:**
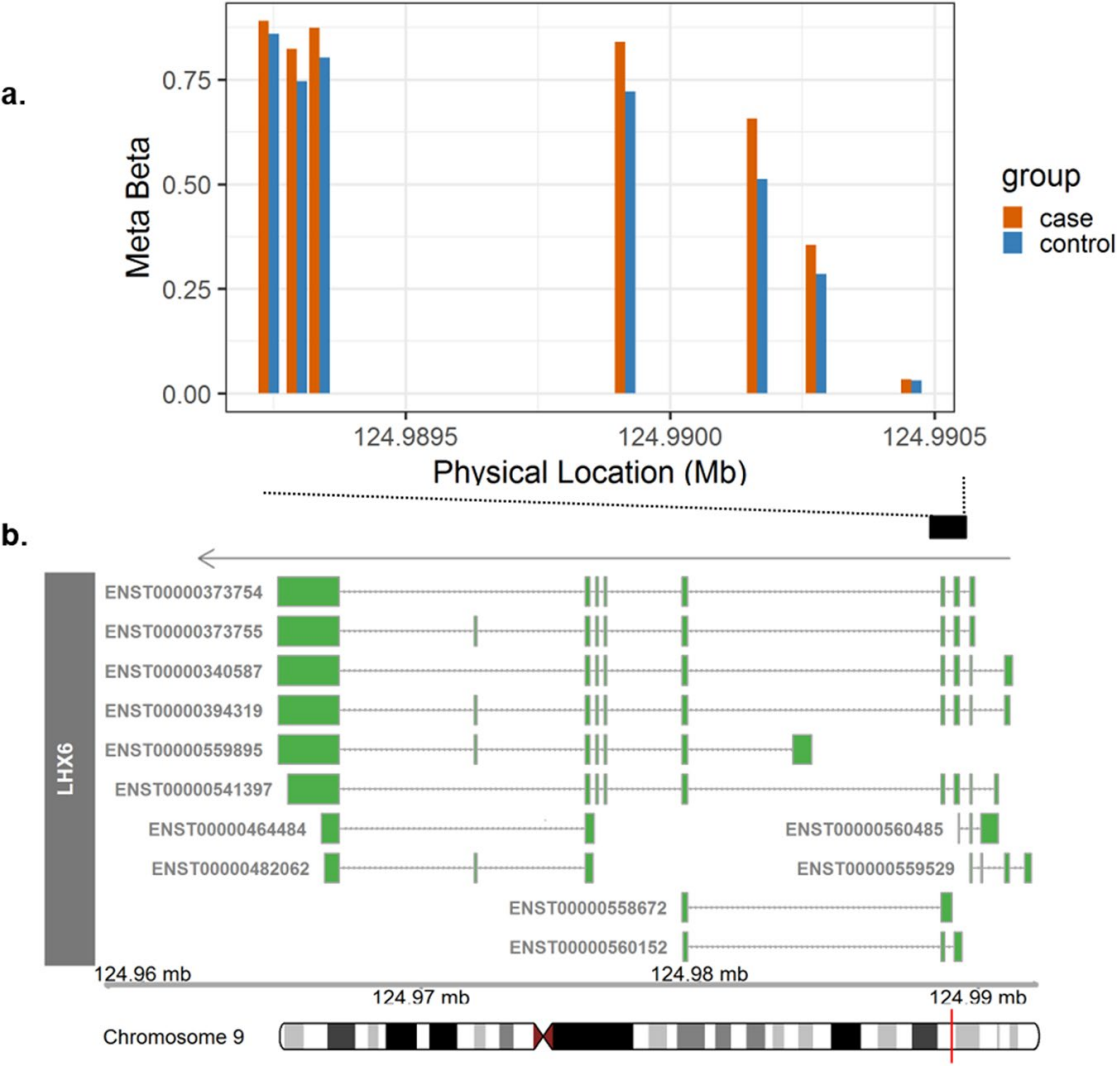
The most statistically significant of 28 differentially methylated regions (DMRs) associated with development of T1D. Average longitudinal methylation from meta-analysis (“Meta Beta”) in T1D cases (orange) and controls (blue) across all seven probes in the DMR on chromosome 9, located near the promoter of LHX6. Structure of LHX6 isoforms from Ensembl are displayed, including: exons (boxes) and introns (lines). The arrow indicates the direction of transcription.

Functional annotation indicated the majority (n=19) of DMRs were located near protein-coding genes. The other 9 DMRs were located closest to sequences of the genome yielding non-coding transcripts, including micro-RNA, long-intergenic non-coding RNA (lincRNA), pseudogene, antisense, and processed transcript. DMRs were most frequently located on chromosome 2 (n = 4), chromosome 8 (n = 4), and chromosome 17 (n = 4). All four DMRs on chromosome 8 were located near *ERICH1-AS1* and *DLGAP2*. The most statistically significant DMR (combined regional adjusted meta p-value = 2.04 × 10^−10^) contained seven probes spanning the TSS, 5′UTR, introns, and coding sequence of the *LHX6* gene (Fig. 4). With average hypermethylation of 7.1% of T1D cases compared to controls, this DMR had the largest effect size, contained the most probes, and spanned the largest sequence of the genome. *LHX6* encodes a transcription factor involved in cell fate regulation during embryogenesis and head development^32^.

### Methylation changes in the natural history of disease

**Figure 5:**
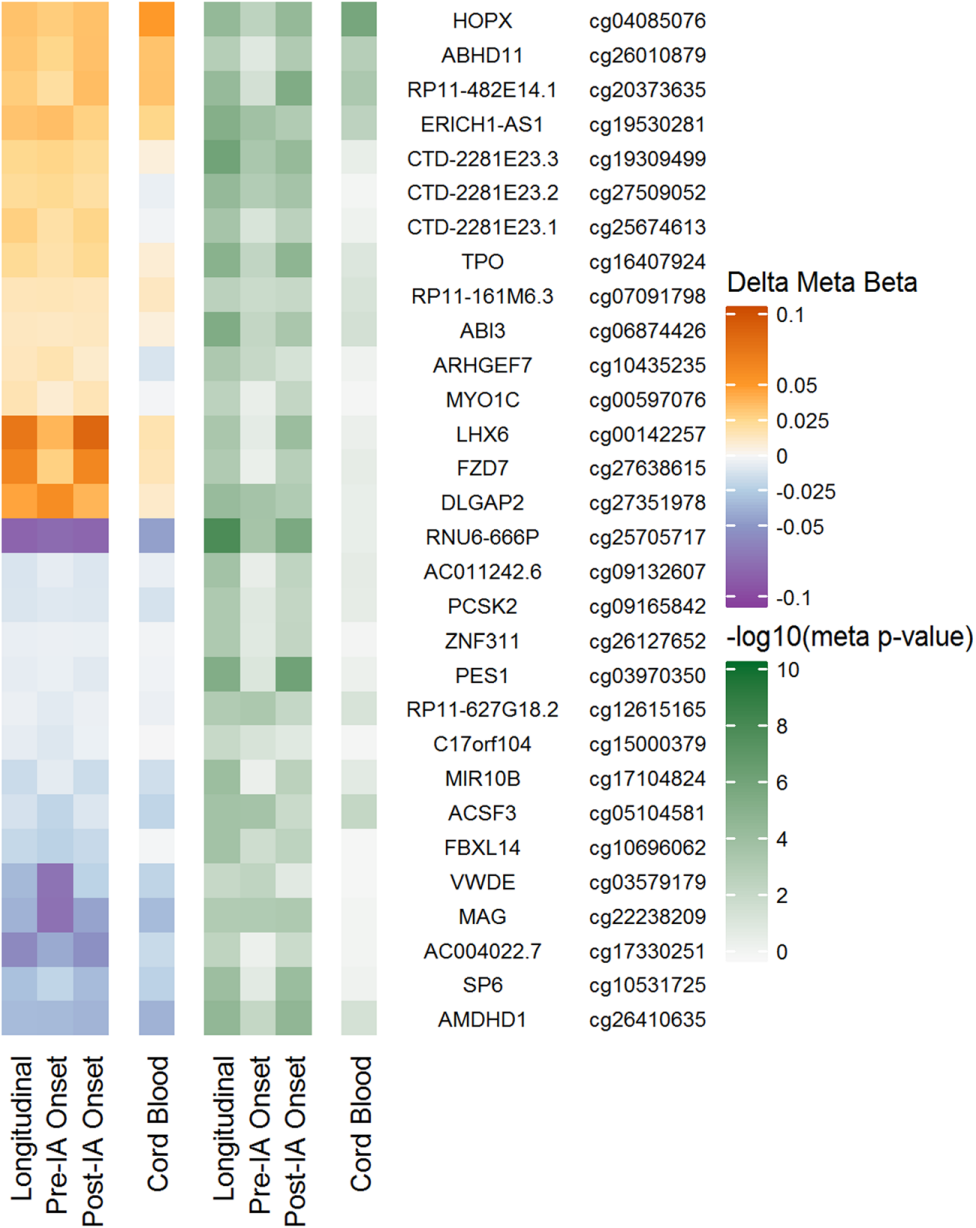
Description of candidate DMPs and DMRs in the natural history of T1D development. Heat map of the methylation difference (delta meta beta) and significance (-log10 meta p-value) comparing T1D cases to controls for longitudinal DMPs and DMRs in cross-sections pre-IA onset, post-IA onset, and in cord blood. Annotation includes nearest gene name and probe name. Cg25705717 and cg19309499 were identified as DMPs; the remaining probes were the most statistically significant from each DMR (n = 28).

Non-genetic factors may differently affect T1D based on phase of autoimmunity, which would not be detectable in the longitudinal model that we employed. Therefore, we characterized the timing of differential methylation in the natural history of the disease by looking at clinically relevant cross-sections pre-IA onset and post-IA onset using the same meta-analysis approach for 450 K and EPIC (see Methods). For example, the pre-IA onset cross-sectional analysis compared the methylation between T1D cases and controls using one sample per subject collected prior to the appearance of autoantibodies in the case (Fig. 1, t = 2 or 3). Given the smaller sample size, only candidate probes were examined in the natural history cross-sectional analyses. Figure 5 shows the direction of effect and unadjusted p-value from all analyses for the two DMPs (cg25705717 and cg19309499) and the most statistically significant probe from each of the 28 DMRs identified from average longitudinal models. All 30 candidates had the same direction of effect (delta meta beta) pre-IA onset and post-IA onset compared to the longitudinal analysis (Supplementary Table S5).

A peak in autoantibody appearance during early childhood suggests the intrauterine or prenatal environment also contributes to T1D risk^33^. In comparing longitudinal candidates to results from a cross-sectional analysis of cord blood available from 37 T1D cases and 35 controls in DAISY, we found 26 candidates had the same direction of methylation effect in cord blood. The remaining four (cg25674613, cg27509052, cg00597076, cg10435235) had slight hypomethylation in cord blood, compared to hypermethylation in the other cross-sections and longitudinally.

Of the 30 candidates, cg04085076 had the largest difference between case and control groups in cord blood. It was consistently hypermethylated in all analyses, with a larger difference between groups in cord blood (5.0%) compared to longitudinally (3.4%), pre-IA onset (3.0%), or post-IA onset (3.5%). The probe was part of a DMR located on chromosome 4 within the TSS, 5′UTR and first exon of *HOPX*. DNA hypermethylation in this area silences expression of *HOPX*^34^, which encodes a homeobox transcription cofactor involved in cellular differentiation, proliferation, and T cell function^35^.

### Methylation differences in EPIC-extension probes

**Figure 6:**
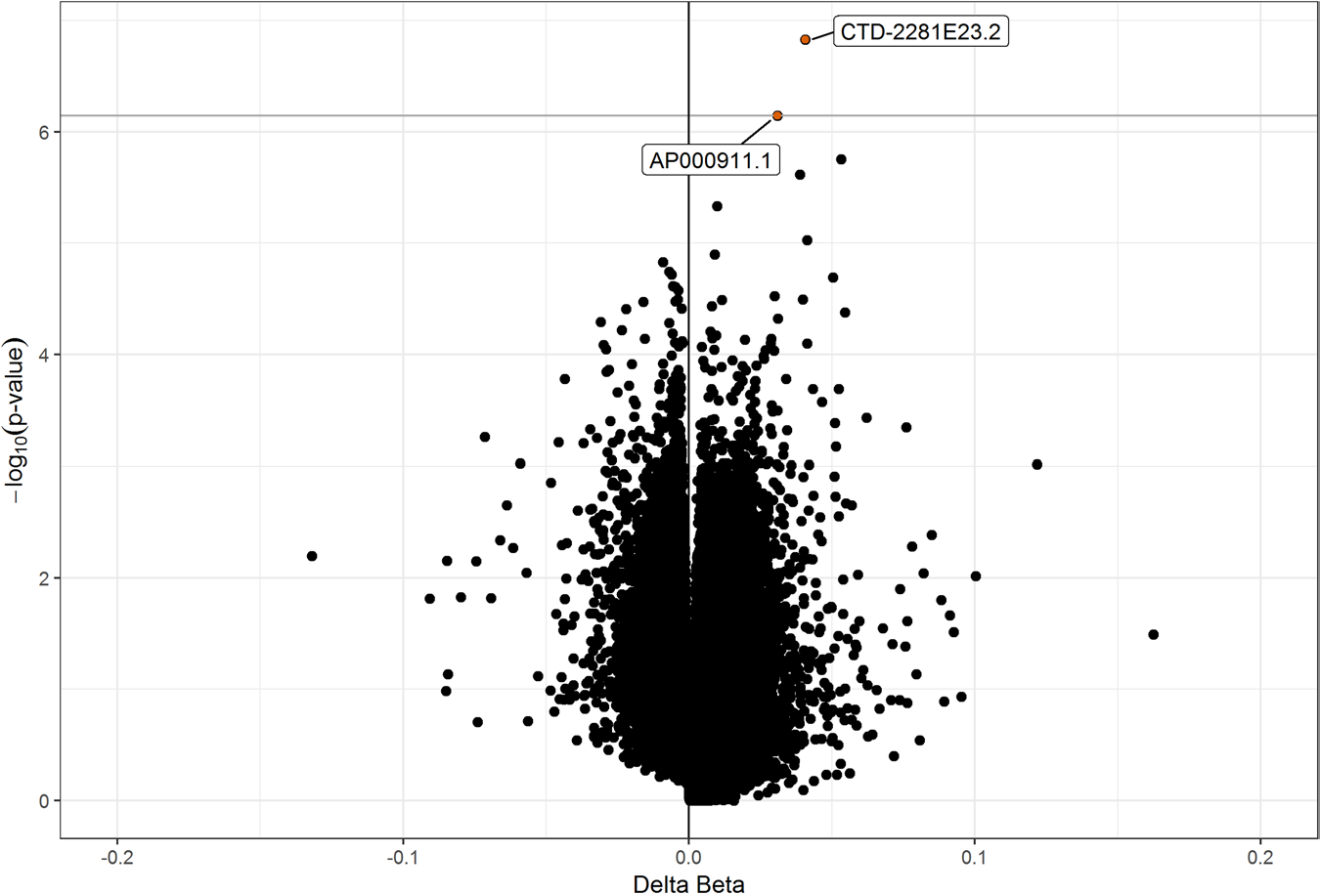
Differentially methylated positions included only on the EPIC platform (EPIC-extension DMPs) longitudinally associated with development of T1D. Volcano plot of results from 215,381 probes that passed pre-processing criteria but were only included on the newer EPIC platform. Delta Beta is the longitudinal average methylation difference between T1D cases compared to controls. Candidate probes with adjusted p-value < 0.1 (solid orange circles) were considered EPIC-extension DMPs and annotated to the gene nearest the probe from Ensembl.

Extended coverage on the EPIC array targeted distal regulatory regions of the genome^36^. There is a growing evidence base that these enhancer regions may determine phenotypic variation for a variety of diseases, including T1D^37^. Therefore, we conducted a second discovery analysis on 215,381 probes on the EPIC platform that were not available on the 450 K array, but that otherwise passed the pre-processing pipeline. We identified 2 candidate EPIC-extension DMPs (adjusted p-value < 0.1, Fig. 6) in longitudinal mixed models adjusted for sex and age. T1D cases had 4.1% average hypermethylation compared to controls at the most statistically significant EPIC-extension DMP (cg24891731, p-value = 1.49 × 10^−7^). It was located near the processed transcript *CTD-2281E23.2* located on chromosome 8 near the gene *ERICH1-AS1*. The other EPIC-extension DMP (cg11405300, p-value = 7.16 × 10^−7^) was characterized by 3.1% hypermethylation in T1D cases compared to controls and was located near *AP000911.1*, a novel miRNA on chromosome 11.

## Discussion

Prior to diagnosis, T1D cases had longitudinally different DNA methylation compared to controls. We identified 10 regions where the rate of methylation change over time differed between groups, and two positions and 28 regions where the average methylation over time differed between groups. These findings expand the current evidence-base on DNA methylation in T1D by establishing that methylation differences are present prior to both IA onset and diabetes diagnosis.

DNA methylation has gained interest as a molecular intermediary in complex disease processes because of its dynamic potential to both direct or reflect transcriptional activity^15,28^. For example, longitudinal hypermethylation near promoters is likely associated with decreased gene expression. Without gene expression data available prior to or at the first detection of autoimmunity in DAISY, we cannot confirm the affect the identified DNA methylation differences may have on gene expression or T1D pathogenesis. However, investigation of previously published and publicly available data suggests several of our candidates may have functional relevance. The DMR probe located within the 5′ UTR of *ACSF3*, cg05104581, was inversely associated with expression of the gene in adult whole blood^38^. Other candidates probes were associated with gene expression in human pancreatic islets^39^, including: cg09118625 (*GNG12*), cg17330251 (*PON1*), cg22238209 (*FFAR1*), and cg26010879 (*CLDN3*). Some candidates we identified were located near transcription factors (*HOPX*, *LHX6*) and miRNA (*MIR10B)*. Functional validation of these candidates should be conducted in other populations.

Using exploratory differential methylation analyses, we identified candidates for future hypothesis-driven investigations into T1D pathogenesis. For example, epigenetic-based parent-of-origin or imprinting effects in T1D have been studied for years, although often inconclusively, in order to explain differential offspring risk by parental T1D^40,41^. We identified differential methylation near several paternally imprinted genes, including DCMRs near *DIRAS3* and *NNAT*, where the rate of methylation change over time differed between groups. In addition, there was a hotspot of average differential methylation on chromosome 8 near the gene *ERICH1-AS1* and the paternally imprinted gene *DLGAP2* that included: one DMP, four DMRs, and one EPIC-extension DMP. *DLGAP2* is primarily studied in neuronal cells and neurological disorders; it may be related to glutamate signaling^42^. These candidates, and those with consistent methylation differences since birth (Fig. 5), are prime targets for exploration of developmental- or parent-of-origin hypotheses.

Inconsistent results of risk factors in T1D development suggests that non-genetic influences may confer different risk by age or autoimmune phase^10^. Our exploration of DNA methylation differences by natural history of the autoimmune phase provided candidates for further investigation into stage-related heterogeneity. We identified several DCMRs where the rate of methylation change with age differed by case-control group. Similarly, our cross-sectional analyses suggested several locations where methylation differences may appear after birth, in the pre-IA onset period (Fig. 5). The observation of directional consistency across analytical frameworks supports our hypothesis that methylation differences generally precede T1D diagnosis, despite differences in subjects and samples included in each analytical framework. A more in-depth analysis of potential age-, autoimmunity- and genotype-related heterogeneity in methylation effects on T1D should also be considered in future work.

Previous T1D methylation studies have identified fewer or no DMPs, but they were conducted among individuals with diagnosed T1D on insulin treatment compared to healthy controls^17–21^. None of the candidate probes with differential methylation in this study were identified as differentially variable between twins in the most recent T1D methylation study by Paul *et al*.^20^. T1D concordance among monozygotic twins increases with time, approaching 65% by age 60 years^43^; however, concordance is often measured at a single point in time in twin studies. Therefore, it is possible differential methylation variability between cases and controls is driven by differences in twin pairs who will remain discordant versus those who will become concordant with time. In checking assumptions of our statistical models (which assume homogeneity of variance between groups), no loci were identified as differentially variable in the main effects average longitudinal methylation analysis (see Methods), in contrast to the thousands identified by Paul *et al*. We speculate these differences may reflect various contributions of epigenetic mechanisms across the disease course, where differential methylation levels may contribute to disease etiology and differential variability post-diagnosis may reflect varying levels of diabetic control, duration of disease, complications, or age among T1D cases.

Use of the DAISY nested case-control study with numerous pre-disease samples per subject is the first attempt to characterize longitudinal methylation patterns prior to T1D diagnosis. Few published human methylation studies of any disease outcome have longitudinal samples available on the same individuals prior to disease. We were able to account for potential confounding variables by adjusting for sex and age, matching on race-ethnicity, estimating cell subtype proportions, and excluding SNP-containing CpGs. We applied rigorous filtering criteria to reduce the number of tests and false positives without sacrificing genome-wide coverage. Despite these efforts, as with many DNA methylation studies^44,45^, identified differences in methylation between groups tended to be near the threshold for genome-wide significance. Replication in other populations and functional validation should be conducted to distinguish true effects from potential false-positives typically generated using array-based technology^46^. While many of the differences between groups we identified were modest in size, small DNA methylation effect sizes have been previously shown to have important and biological impact in various pediatric diseases^47^.

As this was the first exploratory study establishing that DNA methylation precedes T1D diagnosis, the intent was not to inform risk prediction, prognosis, or disease detection. Much remains to be done to make such studies useful for clinical prediction, including using larger sample sizes, replication in other populations, functional validation of candidate sites, etc. However, the current work represents a critical first step toward achieving clinically relevant risk predication models.

We have shown that methylation differences between T1D cases and controls are apparent from birth until pre-diagnosis, including before and after the development of IA. Future work should incorporate functional data to elucidate the potential for these epigenetic changes to contribute to processes involved in T1D pathogenesis.

## Methods

### Study design and population

DAISY has recruited and followed 2,547 Colorado children at high genetic risk of type 1 diabetes since 1993. The screening, follow-up, and case-identification of this well-characterized cohort are described in detail elsewhere^48,49^. Briefly, participants were identified and recruited from population-based newborn screening at St. Joseph’s Hospital in Denver, CO, USA, and from unaffected first-degree relatives of type 1 diabetes patients. Prospective follow-up is ongoing, and has included clinic visits and blood collection at age 9, 15 and 24 months, and annually thereafter until signs of islet autoimmunity appear. DAISY defines IA as the presence of one or more confirmed autoantibody to insulin, GAD65, IA-2, or ZnT8 on two or more consecutive clinic visits. Cases of IA follow an accelerated protocol with clinic visits and blood collection every 3-6 months. Follow-up ends when a participant is diagnosed with diabetes by a physician, defined as typical symptoms of polyuria and/or polydipsia and a random glucose > 11.1 mmol/l or an OGTT with a fasting plasma glucose ≥ 7.0 mmol/l or 2-h glucose > 11.1 mmol/l. The Colorado Multiple Institutional Review Board approved all DAISY study protocols (COMIRB 92-080). Informed consent was obtained from the parents/legal guardians of all children. Assent was obtained from children age 7 years and older. All research was performed in accordance with relevant guidelines/regulations.

This DAISY nested case-control study was selected in January 2015 for investigating epigenetics and other high-dimensional biomarkers in the development of both IA and diabetes endpoints. Cases of T1D were frequency matched to controls based on age at seroconversion to IA, race-ethnicity, and sample availability for five time points of interest in the disease course—e.g., birth, infancy (9 to 16 months of age), the visit just prior to IA (maximum of 2 years prior), the visit at which IA was detected, and the most recent visit prior to T1D diagnosis. Eligible controls were autoantibody- and T1D-free at the age the case was first detected to have IA and remained autoantibody- and T1D-free at the age just prior to T1D diagnosis for the case. A large majority of DAISY study participants were Non-Hispanic White (NHW). No minority race-ethnicity groups were large enough to examine separately, so race-ethnicity was categorized into NHW and Other for matching and analysis. All samples were collected prior to the development of T1D.

For investigating global DNA methylation, subjects were randomly split into two sets, keeping serial samples for each case and their frequency matched controls in the same set. The 450 K set included serial samples for cases and controls, and duplicates for quality control checks. The EPIC set included samples for cases and controls, and replicates from the 450 K set for quality control checks. DAISY was not able to collect cord blood samples on all study children, resulting in a smaller subset of 13 T1D cases and 12 controls in the 450 K and 24 T1D cases and 23 controls in the EPIC with cord blood methylation measures.

### DNA methylation, pre-processing and quality checks

Genomic DNA methylation was profiled in peripheral whole blood using the Infinium HumanMethylation450K Beadchip (Illumina, San Diego, CA, USA, “450 K”). The Infinium HumanMethylation EPIC Beadchip (“EPIC”) was used for the second set due to the technology update at the time of sample processing in late 2015 and early 2016. Identical pre-processing and quality check workflows were performed on both the 450 K and EPIC (Supplementary Fig. S1).

First, data were run through the SeSAMe pipeline^50^ for normalization and quality control (QC) using the sesame package (v1.0.0) in R (v3.5.2). The SeSAMe pipeline follows the general steps of first normalizing the data using the Noob normalization method^51^, then performs non-linear dye bias correction and lastly utilizes a new probe detection above background method (pOOBAH) to help identify failed hybridization to target DNA that pass more traditional QC methods. After normalization and dye bias correction, data were examined for quality on both the probe and sample level using the resulting pOOBAH detection above background values. Arrays with high proportion of failed probes (criteria of 100,000 failed probes for 450 K arrays and 200,000 failed probes for the EPIC arrays) were removed from analyses (450 K = 6, EPIC = 2). Probes which failed to be detected above background in at least 1 sample (SeSAMe’s recommended approach) were removed from subsequent analyses (450 K = 109,956, EPIC = 202,222). Batch effects were adjusted for using ComBat^52^ using plate and row as the batch effect.

Arrays with discordant predicted sex and clinically recorded sex were removed from subsequent analyses (450 K = 5, EPIC = 2). Probes with low range of methylation have poor correlation between 450 K and EPIC arrays^23^, and could lead to increased false positive or false negative results, inflated multiple testing adjustment, and poor replication. Following the methodology of Logue *et al*.^23^, we filtered probes with poor correlation between 36 technical replicates within the 450 K with small range (<0.05 Beta value). Because genetic variation at a CpG site affects methylation levels^53^, we further excluded probes with SNPs in the CpG site from analyses (450 K = 1,244, EPIC = 2,311). The final average longitudinal methylation dataset consisted of 199,243 CpG probes located on the 22 autosomal chromosomes. Probes located on the sex chromosomes were excluded from all analyses. Normalized M-values were used for all subsequent statistical analyses, while β-values (scale of 0–1, proportion methylated) were used for biological interpretation in tables and figures^25^.

### Statistical analyses

Significance for longitudinal analyses was assessed at a false discovery rate adjusted meta p-value < 0.1 using the Benjamini Hochberg method^54^. All results were annotated to the genome using version hg19 from the 450K or EPIC annotation manifest. The datasets generated during and/or analysed during the current study are accessible through GEO Series accession number GSE142512 (https://www.ncbi.nlm.nih.gov/geo/query/acc.cgi?acc=GSE142512).

#### Longitudinal DNA methylation differences

To investigate whether methylation changed at different rates over time between T1D cases and controls, we identified differentially changing methylation positions (DCMPs), using linear mixed models with the following fixed effects: the interaction between T1D case/control status and age, the main effects of T1D case/control status and age as well as sex (treated as a covariate to adjust out). A random intercept term was included to account for repeated measures on subjects using an autoregressive 1 (AR1) structure, which included all of the available visits except cord blood. Regression equations describing the longitudinal models are provided in the Supplemental Material (see Supplementary Methods section). HLA genotype is strongly related to development of T1D, but was not adjusted for due to possible collinearity with the case-control effect. To identify differentially methylated positions (DMPs), the same longitudinal model was employed without the interaction term with age.

Each of the 199,243 probes that survived pre-processing and filtering on both platforms was modeled independently in longitudinal analyses. Methylation difference between T1D cases and controls was estimated as the difference in average beta-values between groups. Statistical models were performed separately by platform, and then combined in a meta-analysis to identify candidate DMPs. Genomic inflation was controlled for using BACON (^55^, v1.10.0) which employs a Gibb Sampling method to estimate an empirical null distribution and designed specifically for epigenome- and transcriptome-wide association studies. An unweighted Stouffer’s method^56^ was employed to perform the meta-analysis. This method incorporates both the p-values and test statistics of the individual model results and returns a meta p-value. This meta p-value was then adjusted for multiple comparisons using Benjamini & Hochberg’s FDR method^54^. A meta percent methylation effect size (meta beta) was calculated by using a simple meta-regression using the individual platforms percent methylation (transformed from the estimated M-value in the original model) case and control estimates.

Hypermethylation indicates cases have higher average methylation at the position; hypo- indicates cases have lower average methylation levels. Candidate positions were annotated to the nearest gene elements and genomic features from Ensembl. Positions that were significant at adjusted meta p-value < 0.1 after multiple comparisons correction were considered DMPs or DCMPs.

Differentially methylated regions (DMRs) and differentially changing methylation regions (DCMRs) were identified from the longitudinal model meta-p-values using the command line tool “comb-p”^26^. A p-value of 0.1 served as a starting place for identifying regions. Then, based on genomic proximity, comb-p combined adjacent positions using a window size of 750 bases and calculated a single p-value for each region with multiple comparison correction. Regional methylation difference was calculated as the average position methylation difference for all probes in the region. Each region was annotated to the nearest gene and CpG island. Comb-p analyses were performed on the meta-analysis p-values for the 199,243 probes that survived pre-filtering and were included on both platforms. Regions that had a combined regional adjusted p-value less than 0.1 and contained more than 1 probe were considered DMRs or DCMRs.

We performed a second average longitudinal methylation analysis using probes included only on the newer EPIC platform (n = 215,381, “EPIC-extended DMP”). Using the same workflow described above, we checked probe and array-level quality, normalized the data using the SeSAME package, filtered probes based on range, corrected for genomic inflation using BACON, and identified longitudinal differences in methylation between cases and controls accounting for sex and age.

#### Analyses of whole blood heterogeneity, differentially variable positions (DVPs)

Unlike previous T1D DNA methylation studies, we did not have access to sorted blood for methylation analyses. Therefore, we investigated the relationship between cell type proportions in whole blood and T1D to establish the potential for cell type proportions to serve as confounding or mediating variables of the main association between DNA methylation and development of T1D. Using the minfi R package, we estimated the proportion of CD4+T cells, CD8+T cells, B cells, granulocytes, and monocytes in each whole blood sample using the whole blood reference set^57,58^. Natural killer cells and other cell types with low prevalence in whole blood were unable to be estimated.

To estimate the effect cell subtype adjustment might have on DMPs, we calculated the absolute change and percent change of the difference in meta beta between T1D case and control groups from average longitudinal mixed models with and without cell type adjustment. Cell type-adjusted mixed models did not converge for all probes used in DMP analyses, resulting in 199,230 probes used for cell-type sensitivity analyses. Percent change was calculated as the difference of estimate with and without cell type adjustment as a ratio of the estimate without cell type adjustment.

Variance in methylation has been previously reported to greatly differ in T1D discordant twins^20^. Therefore, we tested the equality of variance from the longitudinal model in cases and controls to identify differentially variable positions (DVPs) using the mean-trimmed Levene’s test^59^. DVPs could then be compared to previous literature, and their DMP models re-run to allow for unequal variance in methylation between T1D case and control groups.

#### Methylation changes in the natural history of disease

To characterize when methylation differences began to appear in the natural history of the disease, we examined DMPs at pre-IA onset and post-IA onset cross-sections using multivariable linear regression adjusted for sex and age. The pre-IA onset cross-section included the most recent visit prior to diagnosis with IA, which sometimes occurred during infancy (Fig. 1, t = 2 or 3). The post-IA onset cross-section included the most recent visit prior to T1D diagnosis that was at or after the IA visit (Fig. 1, t = 4 or 5). A smaller sample of DAISY subjects (those recruited at birth via newborn screening) had cord blood samples available. Only candidate DMPs (n = 2) or the most statistically significant probe from candidate DMRs (n = 28) were tested independently in these cross-sections, which were a subset of samples included in the longitudinal analyses.

We compared results from the cross-sectional models to the longitudinal models to establish the time course of DNA methylation changes before or after the appearance of islet autoimmunity, and potentially *in utero*. Due to large differences in sample size in each analysis, we emphasized the comparison of direction of effect (hyper- or hypomethylation) in longitudinal versus cross-sectional models, without emphasis on the p-value.

#### Gene ontology and enrichment analyses

We identified gene ontology (GO) molecular functions and biological processes and KEGG pathways that were significantly enriched for probes with adjusted meta p-value < 0.001 (n = 299) using the gometh function of the missMethyl R package^27^. In enrichment analyses, genes annotated to the 299 probes are compared to lists of genes in each curated GO term and KEGG pathway to identify which are statistically over-represented. The missMethyl package included an adjustment for the underlying distribution of probes on the array. Only non-nested, biological process and molecular function GO terms were considered.

## Supplementary information

Supplementary Information.

## Data Availability

The datasets generated during and/or analysed during the current study are accessible through GEO Series accession number GSE142512 (https://www.ncbi.nlm.nih.gov/geo/query/acc.cgi?acc=GSE142512).
