# Supplementary material for: Longitudinal DNA methylation differences precede type 1 diabetes
Source: Sci Rep. 2020 Feb 28;10:3721. doi: 10.1038/s41598-020-60758-0 (PMC7048736; doi:10.1038/s41598-020-60758-0)
Supplement: Supplementary file 1 — Supplementary Information. [file 41598_2020_60758_MOESM1_ESM.pdf]

# Longitudinal DNA methylation differences precede type 1 diabetes

Randi K Johnson, Lauren A Vanderlinden, Fran Dong, Patrick M Carry, Jennifer Seifert, Kathleen Waugh, Hanan Shorrosh, Tasha Fingerlin, Brigitte I. Frohnert, Ivana V Yang, Katerina Kechris, Marian Rewers, Jill M Norris

**Supplementary Figure S1: Data pre-processing and quality control pipeline conducted in parallel on 450K and EPIC.**

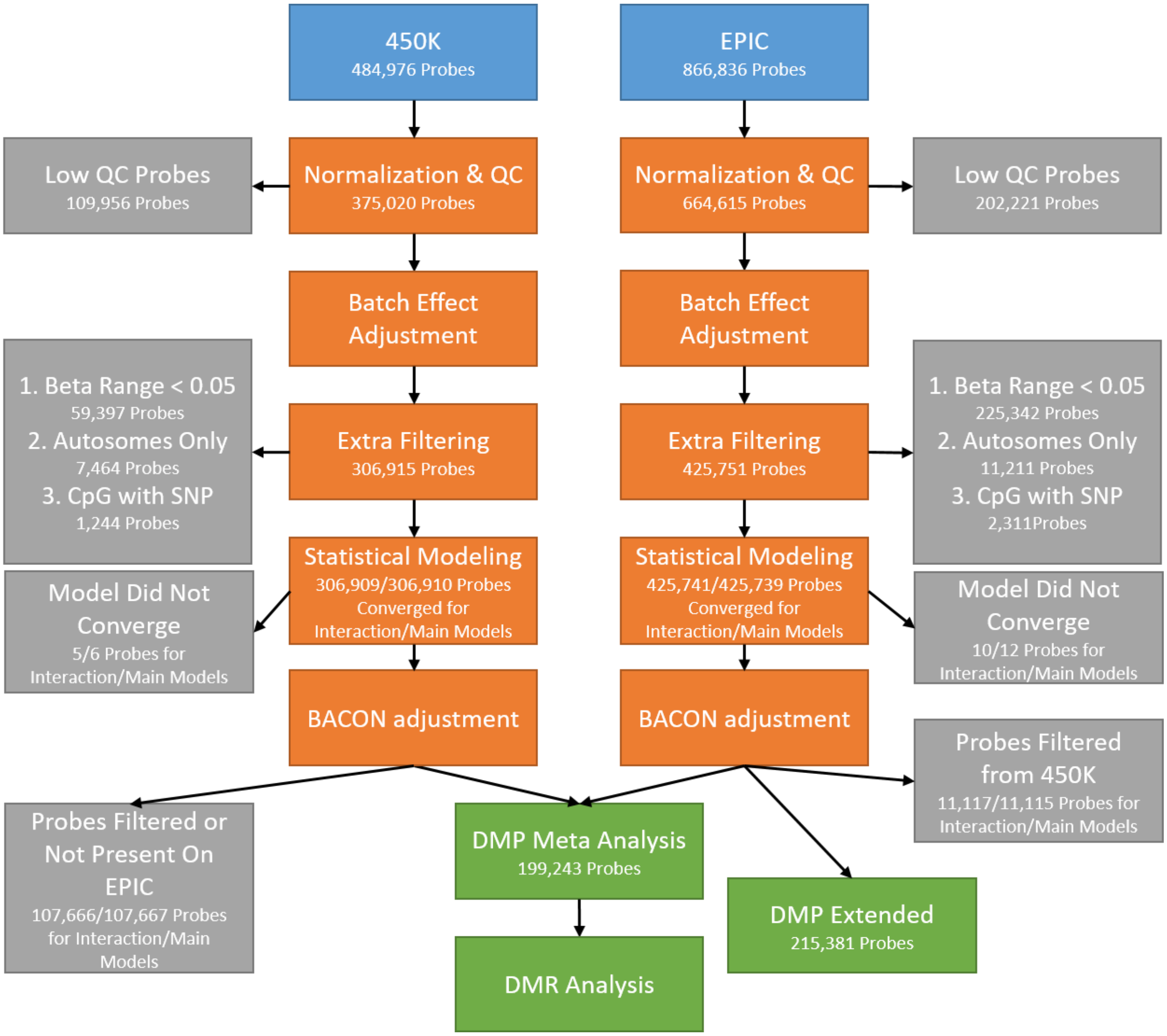

**Supplementary Table S1: Differentially changing methylation regions (DCMRs) where the rate of methylation change with age differed between T1D cases and controls.**

| chr | start | end | n probes | slope control | slope case | adj pvalue | near gene | biotype* |
|---|---|---|---|---|---|---|---|---|
| chr1 | 68512650 | 68513064 | 7 | 0.0042 | 0.0020 | 0.00000672 | DIRAS3 | protein_coding |
| chr6 | 31734192 | 31734581 | 6 | 0.0100 | 0.0073 | 0.0000192 | VWA7 | protein_coding |
| chr1 | 223566447 | 223566795 | 6 | 0.0011 | -0.0010 | 0.0002 | C1orf65 | protein_coding |
| chr14 | 24780404 | 24780735 | 6 | -0.0018 | -0.0045 | 0.000286 | LTB4R, CIDEB | protein_coding |
| chr20 | 36148954 | 36149232 | 12 | 0.0015 | -0.0004 | 0.0008869 | NNAT | protein_coding |
| chr19 | 49223814 | 49224034 | 4 | -0.0011 | -0.0029 | 0.01516 | RASIP1 | protein_coding |
| chr16 | 83171068 | 83171300 | 3 | -0.0077 | -0.0050 | 0.01575 | CTD-3253I12.1 | Antisense |
| chr6 | 48036409 | 48036618 | 5 | 0.0019 | 0.0006 | 0.02318 | PTCHD4 | protein_coding |
| chr7 | 56515666 | 56515847 | 3 | 0.0009 | -0.0009 | 0.02595 | RP13-492C18.2 | Pseudogene |
| chr19 | 36485282 | 36485361 | 3 | 0.0005 | -0.0022 | 0.05705 | SDHAF1 | protein_coding |

**Gene and transcript types defined by Ensembl annotation*

**Supplementary Figure S2: QQplots and genomic inflation factors from average longitudinal differential methylation analyses on 450K and EPIC.**

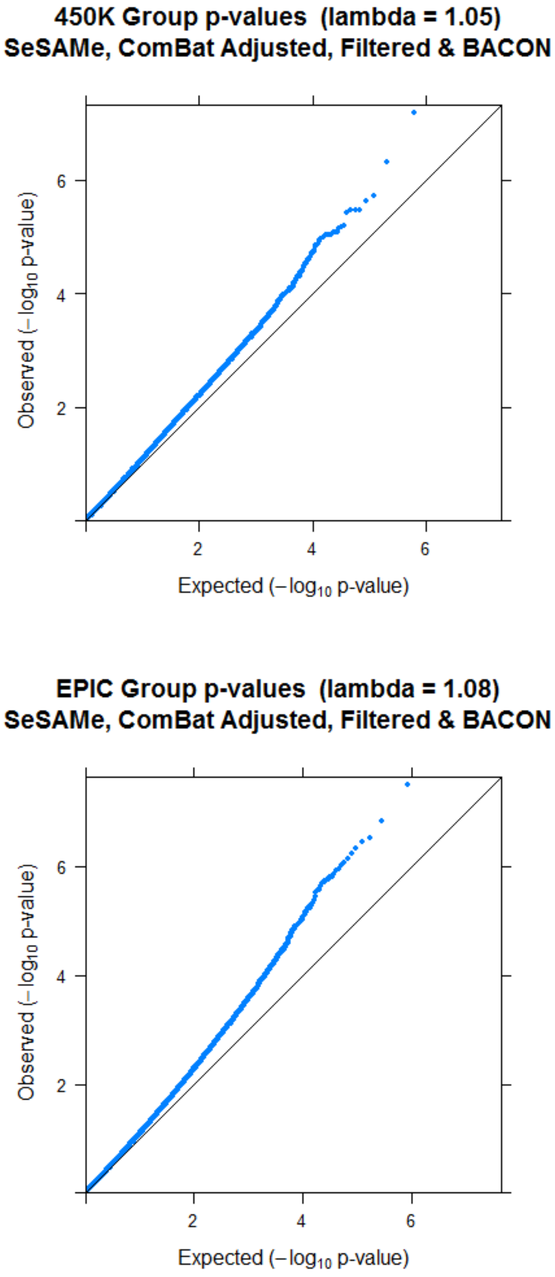

**Supplementary Table S2: Top 10 enriched GO terms in 299 probes with meta p-value < 0.001.**

| Term | Ontology | Number of Genes | Number of Significant Genes | P-value | FDR | ID |
|---|---|---|---|---|---|---|
| isoquinoline alkaloid metabolic process | BP | 2 | 2 | 0.000797 | 1 | GO:0033076 |
| phytoalexin metabolic process | BP | 2 | 2 | 0.000797 | 1 | GO:0052314 |
| transport vesicle | CC | 353 | 12 | 0.000834 | 1 | GO:0030133 |
| calcium-dependent cysteine-type endopeptidase activity | MF | 15 | 3 | 0.001028 | 1 | GO:0004198 |
| calcium-dependent ATPase activity | MF | 3 | 2 | 0.001072 | 1 | GO:0030899 |
| alkaloid metabolic process | BP | 3 | 2 | 0.001181 | 1 | GO:0009820 |
| voltage-gated calcium channel complex | CC | 42 | 5 | 0.001189 | 1 | GO:0005891 |
| troponin C binding | MF | 3 | 2 | 0.001198 | 1 | GO:0030172 |
| oxidoreductase activity, acting on paired donors, with incorporation or reduction of molecular oxygen, reduced pteridine as one donor, and incorporation of one atom of oxygen | MF | 7 | 2 | 0.001357 | 1 | GO:0016714 |
| transport vesicle membrane | CC | 188 | 8 | 0.0015 | 1 | GO:0030658 |

**Supplementary Table S3: Top 10 enriched KEGG pathways in 299 probes with meta p-value < 0.001.**

| Pathway | Number of Genes | Number of Significant Genes | P-value | FDR | ID |
|---|---|---|---|---|---|
| Tyrosine metabolism | 31 | 3 | 0.006197 | 0.820089 | path:hsa00350 |
| Autoimmune thyroid disease | 30 | 3 | 0.00725 | 0.820089 | path:hsa05320 |
| Type II diabetes mellitus | 45 | 4 | 0.007455 | 0.820089 | path:hsa04930 |
| Type I diabetes mellitus | 36 | 3 | 0.014499 | 1 | path:hsa04940 |
| Folate biosynthesis | 25 | 2 | 0.017458 | 1 | path:hsa00790 |
| Dopaminergic synapse | 127 | 5 | 0.033588 | 1 | path:hsa04728 |
| Cell adhesion molecules (CAMs) | 128 | 5 | 0.035771 | 1 | path:hsa04514 |
| Rheumatoid arthritis | 81 | 3 | 0.041011 | 1 | path:hsa05323 |
| Graft-versus-host disease | 30 | 2 | 0.049472 | 1 | path:hsa05332 |
| Allograft rejection | 29 | 2 | 0.050154 | 1 | path:hsa05330 |

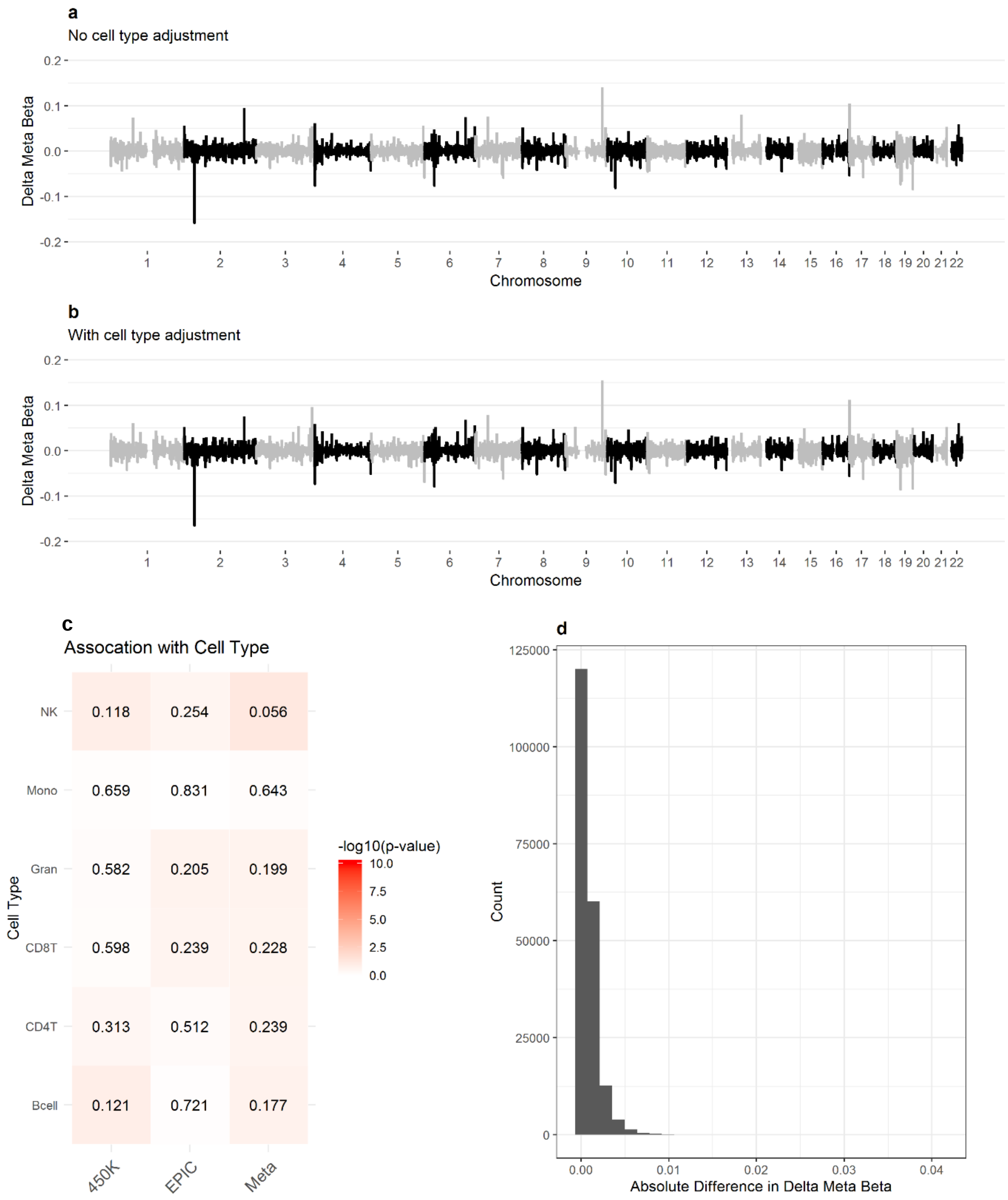


**Supplementary Figure S3: Sensitivity analysis adjusting for cell type proportions in longitudinal meta-analysis for 199,230 probes.** a-b) Delta meta beta (methylation difference) between T1D cases and controls with and without cell type adjustment. c) P-value heat map of longitudinal associations of cell types with T1D from linear mixed models adjusted for sex and age, by panel and meta-analysis. d) Absolute difference (cell type adjusted – non-adjusted) in delta meta beta (median = $5.4 \times 10^{-4}$, IQR: $2.4 \times 10^{-4}$ to $1.1 \times 10^{-3}$).

**Supplementary Table S4: Differentially methylated regions associated with development of T1D.**

| DMR | chr | start | end | n probes | pct hyper | avg beta | adj pvalue | near gene | biotype |
|---|---|---|---|---|---|---|---|---|---|
| 1 | chr9 | 124989241 | 124990457 | 7 | 100 | 0.071259405 | 2.04E-10 | LHX6 | protein_coding |
| 2 | chr4 | 57547347 | 57548094 | 5 | 100 | 0.032198331 | 6.4E-09 | HOPX | protein_coding |
| 3 | chr12 | 96350519 | 96350796 | 5 | 0 | -0.021779598 | 0.000000587 | AMDHD1 | protein_coding |
| 4 | chr17 | 47287410 | 47287578 | 4 | 100 | 0.012087365 | 0.00000284 | ABI3 | protein_coding |
| 5 | chr8 | 1113291 | 1113433 | 2 | 100 | 0.03731453 | 0.0000198 | ERICH1-AS1 | antisense |
| 6 | chr2 | 43903227 | 43903651 | 4 | 0 | -0.013392819 | 0.0000402 | AC011242.6 | pseudogene |
| 7 | chr2 | 177014849 | 177015126 | 6 | 0 | -0.013086321 | 0.000095 | MIR10B | miRNA |
| 8 | chr12 | 1725788 | 1726077 | 5 | 0 | -0.012564324 | 0.0001327 | FBXL14 | protein_coding |
| 9 | chr8 | 1649868 | 1650173 | 3 | 100 | 0.026054591 | 0.0002919 | DLGAP2 | protein_coding |
| 10 | chr8 | 1012324 | 1012466 | 2 | 100 | 0.013360668 | 0.0005535 | CTD-2281E23.2 | processed_transcript |
| 11 | chr6 | 28973328 | 28973521 | 6 | 0 | -0.004953171 | 0.001018 | ZNF311 | protein_coding |
| 12 | chr2 | 202901352 | 202901471 | 3 | 100 | 0.070727636 | 0.002206 | FZD7 | protein_coding |
| 13 | chr8 | 1273604 | 1273857 | 4 | 100 | 0.021114917 | 0.005035 | CTD-2281E23.1 | lincRNA |
| 14 | chr22 | 31002892 | 31003148 | 7 | 0 | -0.003539409 | 0.005239 | TCN2, PES1 | protein_coding |
| 15 | chr7 | 94953810 | 94954203 | 5 | 0 | -0.041959065 | 0.006206 | AC004022.7 | pseudogene |
| 16 | chr17 | 1395864 | 1396124 | 5 | 100 | 0.016914269 | 0.007037 | MYO1C | protein_coding |
| 17 | chr2 | 1452260 | 1452368 | 2 | 100 | 0.018378147 | 0.00868 | TPO | protein_coding |
| 18 | chr18 | 19756877 | 19757023 | 3 | 0 | -0.005802175 | 0.01757 | RP11-627G18.2 | antisense |
| 19 | chr10 | 3466795 | 3466853 | 2 | 100 | 0.028986776 | 0.0195 | RP11-482E14.1 | lincRNA |
| 20 | chr16 | 1060367 | 1060559 | 3 | 100 | 0.015585702 | 0.02279 | RP11-161M6.3 | lincRNA |
| 21 | chr19 | 35800589 | 35800744 | 2 | 0 | -0.027056551 | 0.02335 | MAG | protein_coding |
| 22 | chr7 | 12443880 | 12444116 | 5 | 0 | -0.027820051 | 0.03191 | VWDE | protein_coding |
| 23 | chr13 | 111956623 | 111956696 | 2 | 100 | 0.013535115 | 0.04095 | ARHGEF7 | protein_coding |
| 24 | chr17 | 45924888 | 45925061 | 2 | 0 | -0.022103851 | 0.04459 | SP6 | protein_coding |
| 25 | chr7 | 73157217 | 73157384 | 2 | 100 | 0.036716466 | 0.0455 | ABHD11 | protein_coding |
| 26 | chr20 | 17296317 | 17296451 | 2 | 0 | -0.011337132 | 0.04911 | PCSK2 | protein_coding |
| 27 | chr17 | 42733527 | 42733699 | 5 | 0 | -0.011711252 | 0.07489 | C17orf104 | protein_coding |

| | | | | | | | | | |
|---|---|---|---|---|---|---|---|---|---|
| 28 | chr16 | 89164953 | 89164991 | 2 | 0 | -0.011399305 | 0.09162 | ACSF3 | protein_coding |

**Supplementary Table S5: Longitudinal and cross-sectional results for candidate probes associated with the development of T1D (DMPs or within DMRs).**

| probe | type | chr | pos | Long FDR | Long pvalue | PreSV pvalue | PostSV pvalue | CB pvalue | Long Beta | PreSV Beta | PostSV Beta | CB Beta | near gene | biotype |
|---|---|---|---|---|---|---|---|---|---|---|---|---|---|---|
| cg25705717 | DMP | chr10 | 27608719 | 3.92E-03 | 1.97E-08 | 2.24E-04 | 2.58E-06 | 2.23E-01 | -0.081 | -0.076 | -0.079 | -0.045 | RNU6-666P | snRNA |
| cg19309499 | DMP | chr8 | 1150488 | 8.89E-02 | 8.92E-07 | 3.62E-04 | 4.22E-05 | 2.17E-01 | 0.025 | 0.026 | 0.023 | 0.007 | CTD-2281E23.3 | lincRNA |
| cg03970350 | DMR | chr22 | 31003010 | 1.99E-01 | 5.00E-06 | 5.81E-02 | 7.90E-07 | 3.26E-01 | -0.006 | -0.005 | -0.008 | -0.003 | PES1 | protein_coding |
| cg06874426 | DMR | chr17 | 47287526 | 1.99E-01 | 4.11E-06 | 4.74E-03 | 3.49E-04 | 2.46E-02 | 0.013 | 0.012 | 0.013 | 0.007 | ABI3 | protein_coding |
| cg19530281 | DMR | chr8 | 1113432 | 2.24E-01 | 6.74E-06 | 1.36E-04 | 7.53E-04 | 2.54E-03 | 0.034 | 0.036 | 0.028 | 0.026 | ERICH1-AS1 | antisense |
| cg16407924 | DMR | chr2 | 1452260 | 2.52E-01 | 1.07E-05 | 4.04E-03 | 1.89E-05 | 6.54E-02 | 0.023 | 0.018 | 0.024 | 0.008 | TPO | protein_coding |
| cg04085076 | DMR | chr4 | 57547579 | 2.73E-01 | 3.61E-05 | 2.59E-03 | 3.66E-05 | 1.65E-06 | 0.034 | 0.030 | 0.035 | 0.050 | HOPX | protein_coding |
| cg26410635 | DMR | chr12 | 96350720 | 2.73E-01 | 2.73E-05 | 5.31E-03 | 2.45E-05 | 2.82E-02 | -0.033 | -0.033 | -0.036 | -0.037 | AMDHD1 | protein_coding |
| cg27509052 | DMR | chr8 | 1012324 | 2.73E-01 | 3.81E-05 | 8.47E-04 | 2.10E-04 | 6.87E-01 | 0.022 | 0.024 | 0.020 | -0.005 | CTD-2281E23.2 | processed_transcript |
| cg20373635 | DMR | chr10 | 3466795 | 2.75E-01 | 4.37E-05 | 2.43E-02 | 4.41E-06 | 4.51E-04 | 0.030 | 0.021 | 0.036 | 0.034 | RP11-482E14.1 | lincRNA |
| cg27351978 | DMR | chr8 | 1650172 | 2.80E-01 | 5.48E-05 | 2.06E-04 | 6.90E-04 | 2.23E-01 | 0.046 | 0.058 | 0.039 | 0.011 | DLGAP2 | protein_coding |
| cg10531725 | DMR | chr17 | 45924888 | 3.68E-01 | 8.77E-05 | 1.21E-01 | 6.71E-05 | 4.23E-01 | -0.031 | -0.020 | -0.033 | -0.022 | SP6 | protein_coding |
| cg17104824 | DMR | chr2 | 177014959 | 3.68E-01 | 8.27E-05 | 2.94E-01 | 1.97E-03 | 1.23E-01 | -0.017 | -0.007 | -0.016 | -0.015 | MIR10B | miRNA |
| cg05104581 | DMR | chr16 | 89164990 | 4.68E-01 | 1.64E-04 | 2.04E-04 | 8.44E-03 | 5.24E-03 | -0.013 | -0.020 | -0.010 | -0.020 | ACSF3 | protein_coding |
| cg09132607 | DMR | chr2 | 43903582 | 4.68E-01 | 1.71E-04 | 2.07E-01 | 3.14E-03 | 1.53E-01 | -0.011 | -0.006 | -0.010 | -0.005 | AC011242.6 | pseudogene |
| cg10696062 | DMR | chr12 | 1726028 | 4.68E-01 | 1.71E-04 | 1.42E-02 | 2.59E-03 | 8.09E-01 | -0.019 | -0.022 | -0.018 | -0.001 | FBXL14 | protein_coding |
| cg25674613 | DMR | chr8 | 1273808 | 5.06E-01 | 2.31E-04 | 4.29E-02 | 2.08E-03 | 4.11E-01 | 0.028 | 0.019 | 0.026 | -0.002 | CTD-2281E23.1 | lincRNA |
| cg00142257 | DMR | chr9 | 124990276 | 5.46E-01 | 3.32E-04 | 1.46E-01 | 7.09E-05 | 2.91E-01 | 0.072 | 0.039 | 0.083 | 0.017 | LHX6 | protein_coding |

| | | | | | | | | | | | | | | |
|---|---|---|---|---|---|---|---|---|---|---|---|---|---|---|
| cg10435235 | DMR | chr13 | 111956623 | 5.82E-01 | 4.81E-04 | 6.22E-03 | 3.10E-02 | 4.23E-01 | 0.014 | 0.017 | 0.010 | -0.011 | ARHGEF7 | protein_coding |
| cg26127652 | DMR | chr6 | 28973426 | 5.82E-01 | 5.06E-04 | 9.53E-02 | 4.11E-03 | 6.54E-01 | -0.005 | -0.005 | -0.005 | -0.002 | ZNF311 | protein_coding |
| cg09165842 | DMR | chr20 | 17296317 | 5.86E-01 | 5.18E-04 | 8.95E-02 | 4.77E-03 | 1.71E-01 | -0.011 | -0.009 | -0.010 | -0.013 | PCSK2 | protein_coding |
| cg27638615 | DMR | chr2 | 202901352 | 6.07E-01 | 6.39E-04 | 2.53E-01 | 1.38E-03 | 1.74E-01 | 0.062 | 0.028 | 0.062 | 0.016 | FZD7 | protein_coding |
| cg12615165 | DMR | chr18 | 19756877 | 6.14E-01 | 7.23E-04 | 4.17E-04 | 5.46E-03 | 3.69E-02 | -0.004 | -0.008 | -0.005 | -0.004 | RP11-627G18.2 | antisense |
| cg22238209 | DMR | chr19 | 35800743 | 6.14E-01 | 7.15E-04 | 6.88E-04 | 5.21E-04 | 5.88E-01 | -0.037 | -0.073 | -0.044 | -0.033 | MAG | protein_coding |
| cg26010879 | DMR | chr7 | 73157217 | 6.72E-01 | 1.15E-03 | 8.63E-02 | 6.58E-04 | 1.21E-03 | 0.033 | 0.026 | 0.035 | 0.034 | ABHD11 | protein_coding |
| cg07091798 | DMR | chr16 | 1060367 | 7.09E-01 | 2.15E-03 | 9.49E-03 | 6.28E-03 | 3.89E-02 | 0.015 | 0.015 | 0.014 | 0.013 | RP11-161M6.3 | lincRNA |
| cg00597076 | DMR | chr17 | 1395880 | 7.15E-01 | 2.28E-03 | 2.29E-01 | 4.70E-03 | 7.70E-01 | 0.016 | 0.009 | 0.016 | -0.002 | MYO1C | protein_coding |
| cg17330251 | DMR | chr7 | 94953956 | 7.38E-01 | 3.00E-03 | 3.31E-01 | 9.72E-03 | 6.18E-01 | -0.058 | -0.041 | -0.054 | -0.017 | AC004022.7 | pseudogene |
| cg03579179 | DMR | chr7 | 12444095 | 7.87E-01 | 6.55E-03 | 3.13E-03 | 1.09E-01 | 8.35E-01 | -0.034 | -0.072 | -0.022 | -0.021 | VWDE | protein_coding |
| cg15000379 | DMR | chr17 | 42733662 | 7.95E-01 | 7.05E-03 | 3.90E-02 | 9.79E-02 | 7.08E-01 | -0.006 | -0.010 | -0.004 | -0.001 | C17orf104 | protein_coding |

**Supplementary Methods: Multivariable regression equations**

Differentially changing methylation positions (DCMPs)

$$DNA\ methylation_{ij} = \beta_0 + \beta_{case} * caseStatus_{ij} + \beta_{age} * age_{ij} + \beta_{case*age} * caseStatus_{ij} * age_{ij} + \beta_{sex} * sex_{ij} + \varepsilon_{ij}$$

Where:

- $DNA\ methylation_{ij}$ is the M-value DNA methylation for the $j^{th}$ of $n_i$ observations in the $i^{th}$ person
- $\beta_0, \ldots.\beta_{case}$ are the fixed-effect coefficients (including the intercept), which is identical for all subjects
- $caseStatus_{ij}$ is the fixed-effect case status variable (either case or control) for observation *j* in subject *i*
- $age_{ij}$ is the fixed-effect age variable (measured out to reflect the specific day) for observation *j* in subject *i*
- $caseStatus_{ij} * age_{ij}$ is the fixed-effect interaction between the case status and age variables for observation *j* in subject *i*
- $sex_{ij}$ is the fixed-effect sex variable (either male or female) for observation *j* in subject *i*
- $\varepsilon_{ij} = \phi\varepsilon_{ij-1} + Z_{ij}, \quad Z_{ij} \sim iid\ N(0, \sigma_Z^2)$ where $\varepsilon_{ij}$ is the error for observation *j* in subject *i* and $\phi$ is the autocorrelation coefficient (fitting an autoregressive order 1 covariance structure)

Differential methylation positions (DMPs)

$$DNA\ methylation_{ij} = \beta_0 + \beta_{case} * caseStatus_{ij} + \beta_{age} * age_{ij} + \beta_{sex} * sex_{ij} + \varepsilon_{ij}$$

Where:

- $DNA\ methylation_{ij}$ is the M-value DNA methylation for the $j^{th}$ of $n_i$ observations in the $i^{th}$ person
- $\beta_0, \ldots.\beta_{case}$ are the fixed-effect coefficients (including the intercept), which is identical for all subjects
- $caseStatus_{ij}$ is the fixed-effect case status variable (either case or control) for observation *j* in subject *i*
- $age_{ij}$ is the fixed-effect age variable (measured out to reflect the specific day) for observation *j* in subject *i*
- $sex_{ij}$ is the fixed-effect sex variable (either male or female) for observation *j* in subject *i*
- $\varepsilon_{ij} = \phi\varepsilon_{ij-1} + Z_{ij}, \quad Z_{ij} \sim iid\ N(0, \sigma_Z^2)$ where $\varepsilon_{ij}$ is the error for observation *j* in subject *i* and $\phi$ is the autocorrelation coefficient (fitting an autoregressive order 1 covariance structure)
